# Enhancing attachment-based aspects of PCIT for young children with a history of maltreatment

**DOI:** 10.3389/fpsyg.2023.1229109

**Published:** 2023-11-03

**Authors:** Kristine Belanger, Hannah Gennis, Nicole Ottenbreit, Nicole Racine

**Affiliations:** ^1^Child Abuse Service, Alberta Children’s Hospital, Calgary, AB, Canada; ^2^Luna Child and Youth Advocacy Centre, Calgary, AB, Canada; ^3^Hospital for Sick Children, Toronto, ON, Canada; ^4^School of Psychology, University of Ottawa, Ottawa, ON, Canada; ^5^Children’s Hospital of Eastern Ontario Research Institute, Ottawa, ON, Canada

**Keywords:** child maltreatment, parenting, parent child interaction therapy, behavior difficulties, circle of security parenting

## Abstract

Disruptive behavior difficulties, such as aggression, non-compliance, and emotional outbursts, are common among children exposed to maltreatment. Parent–Child Interaction Therapy (PCIT) is an effective parenting intervention for addressing child behavior difficulties, however, treatment retention and engagement among parents remain a concern in the clinical setting. This paper describes how the delivery of an intervention that teaches attachment theory concepts (Circle of Security-Parenting, COS-P) prior to PCIT can increase engagement and retention among parents of maltreated children and inform new coaching practices. A detailed description of how to extend and integrate COS-P concepts with PCIT for maltreated families using specific strategies is provided. Recommendations, limitations, and next steps for research are presented.

## 1. Introduction

Young children who are exposed to trauma, particularly maltreatment, are at risk of developing a host of negative outcomes throughout the lifespan (Toth and Cicchetti, 2013; Gardner et al., 2019). As a result of exposure to frightening, violent, and upsetting experiences, children exposed to maltreatment experience disruptions in their developmental, emotional, and social skills that lead to behavior difficulties, such as aggression, non-compliance, and emotional outbursts (Racine et al., 2021). Behavior difficulties in young children occur in the context of the parent–child relationship, which is also the primary mechanism by which improvements in child outcomes can be achieved (Valentino, 2017). As such, parenting interventions that increase parenting skills and improve the quality of the parent–child relationship have been identified as the primary approach for addressing child behavior difficulties for maltreated children.

Although several interventions can be used to address behavioral difficulties in children who have been maltreated, Parent–Child Interaction Therapy (PCIT) has been one of the most robustly studied interventions. A recent systematic review of 40 studies of families presenting with child maltreatment found that PCIT is associated with improvements in both child and parent outcomes, including parenting stress, child behavior problems, child trauma symptoms, parent mental health difficulties, and negative parenting strategies (Warren et al., 2022). However, a significant limitation of PCIT noted in the literature has been high attrition rates, with rates as high as 71% (Phillips et al., 2008; Lyon and Budd, 2010; Danko et al., 2016; Lieneman et al., 2019). A recent systematic review found that the average attrition rate among families experiencing maltreatment was 39.3% with a range of 5–71% (Warren et al., 2022). Historically, families referred by child welfare display higher rates of attrition (Campbell et al., 2023). For example, a recent study reported a PCIT graduation rate of 17.8% among children in foster care (Onovbiona et al., 2023), pointing to high rates of attrition among families presenting with maltreatment.

Skoranski et al. (2022) examined the pattern of attrition at different phases in treatment when families in the child welfare system were offered PCIT (Skoranski et al., 2022). Notably, 36% of the parents dropped out prior to engaging in PCIT and these parents tended to: (1) endorse beliefs that they had little control over their children’s behaviors and (2) demonstrate physiological signs of distress during a clean-up task. Taken together, these patterns of attrition among families presenting with maltreatment suggest that parents of maltreated children may benefit from support to better understand their role in shifting their child’s behavior difficulties. In addition, the findings support the role of parental distress in attrition rates and highlight the need to intervene at this level. Specifically, parents of maltreated children may require additional supports to engage in trusting relationships and manage emotions and responses because of their own traumatic childhood experiences (Racine et al., 2021). Although PCIT offers effective strategies to improve child behavior difficulties, there is a need to consider how the intervention can be enhanced to bolster caregiver participation and engagement.

The Circle of Security – Parenting (COS-P) program provides parents with an attachment-based framework to understand their role in shifting their child’s behavior as well as emotion regulation strategies for dealing with child behavior responses (Marvin et al., 2002; Cooper et al., 2009; Powell et al., 2014; Woodhouse et al., 2018). COS-P engages parents by providing them with knowledge about why children are demonstrating challenging behaviors and how their relationship with their child is a vehicle for addressing these concerns. COS-P teaches parents to view disruptive behavior as a miscue of an underlying attachment need. A miscue refers to the child engaging in one behavior (e.g., aggression) in favor of more adaptive behavior (e.g., requesting emotional support directly). Without this knowledge, the child’s need for emotional support is overshadowed by the aggressive behavior and results in negative attributions toward the child. At first, the parent learns to decode disruptive and aggressive behavior as an underlying need that requires parental support and then works toward creating the relational conditions required for a miscued attachment need to be expressed directly. Relational conditions refer to qualities of the parent–child relationship, such as a parent being open to emotion, a parent being emotionally available, or a parent being able to take charge when needed, that are necessary for a child to express an attachment need directly rather than miscue (i.e., behave aggressively). Through the COS-P intervention, attachment needs, and the miscuing process are normalized, and parents learn that they may contribute to the evolution of the miscues. As such, COS-P helps parents to understand why their relationship may need to change (i.e., miscuing attachment needs can be highly problematic for child and parent) and what needs to change for their children to communicate their needs more directly and adaptively.

**Figure 1:**
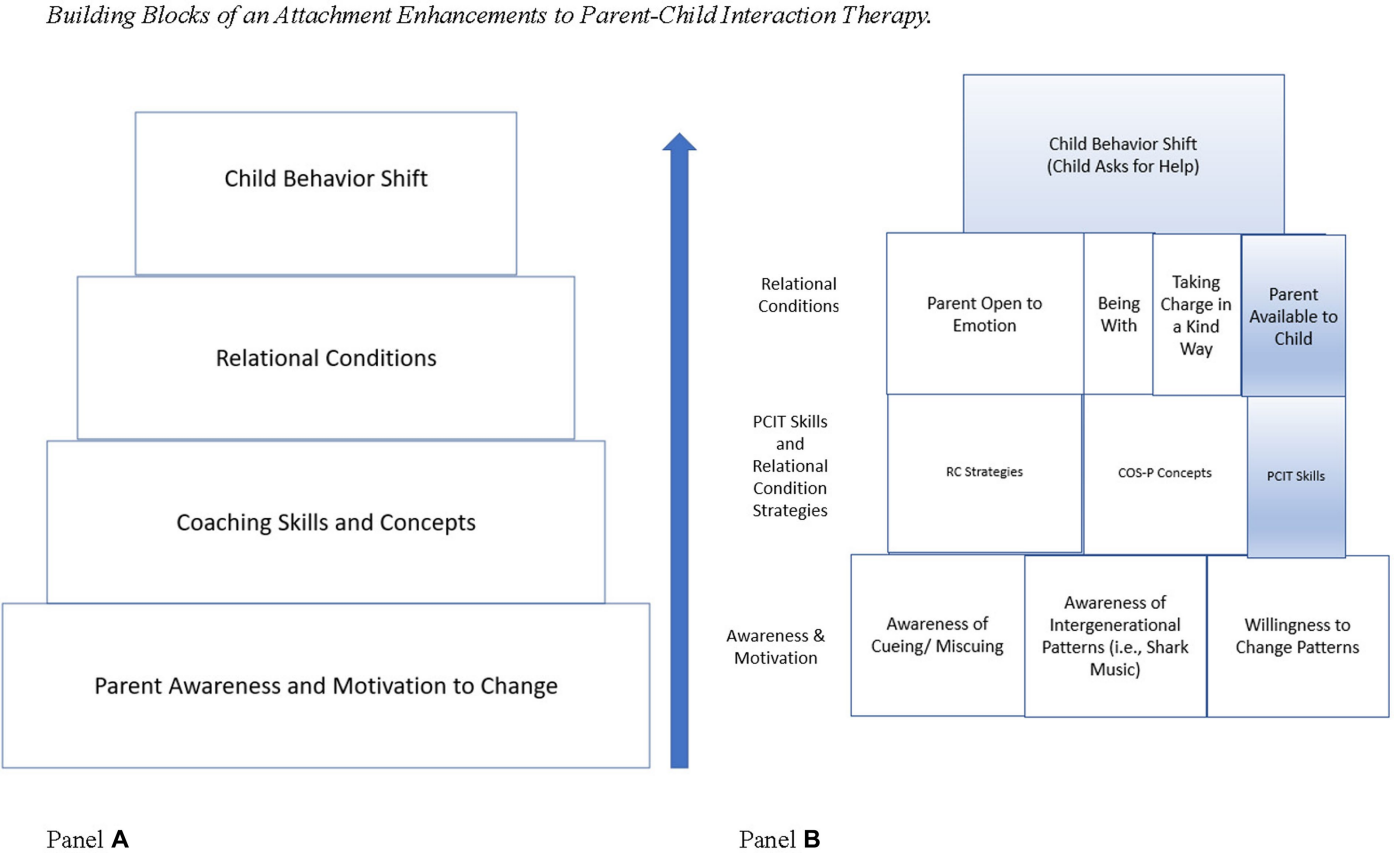
Panel **(A)** depicts the series of conceptual building blocks that must be established before a behavioral shift can occur for the child (top row). When PCIT is modified to include attachment-based enhancements, first the parent must gain an awareness of relational patterns and a motivation to change through the COS-P intervention. Next, through coaching using both PCIT and relational condition strategies, the parent develops the skills they need to establish the relational conditions necessary for the child’s behavior to shift. Panel **(B)** depicts the components of the COS-P and PCIT interventions. The blocks shaded in blue represent those that are present in PCIT alone and the blocks in white represent the additional components of COS-P and the attachment-based modifications.

COS-P provides parents with a framework to understand their child’s behavior. Specifically, COS-P addresses the underlying distress associated with the child’s miscues. COS-P can also increase motivation for parents by providing a framework for *why* and *how* the intervention works which has been shown to be an important component for treatment engagement (Morawska and Sanders, 2006). Thus, pairing COS-P strategies with the effectiveness of PCIT has the potential to increase engagement in the intervention and retention over time for families who have experienced maltreatment. Indeed, COS-P has been used with substance-involved, maltreating caregiver and other high-risk populations and has shown improvements in parenting behavior from pre-to post-intervention (Gerdts-Andresen, 2021; Zimmer-Gembeck et al., 2022) Specifically, participation in COS-P reduces parental stress and enhances parenting self-efficacy and parenting skills in these high-risk populations. Furthermore, enhancing PCIT with attachment-based strategies may more rapidly shift the child’s need to miscue and ultimately increase the likelihood of a shift in the child’s behavior (see Figure 1, Panel **A**).

Enhancements and adaptations to PCIT, while maintaining the core components of the intervention, have been undertaken previously (Eyberg, 2005) with some focused on maltreated children. Time-limited adaptations have been developed and evaluated for families involved with Child Welfare (Thomas and Zimmer-Gembeck, 2012). Further, adaptations for children and families who have experienced trauma, including psychoeducation related to trauma, have also been documented (Gurwitch and Warner-Metzger, 2022). Therefore, previous work has identified a need to tailor PCIT for families presenting with maltreatment. Intervening at the parental level to engage and retain parents in PCIT may prove to be another avenue to supporting these families in PCIT.

### 1.1. Parent child interaction therapy for maltreated children

PCIT was initially developed for families of children (ages 2–7 years) with severe behavioral difficulties such as defiance, excessive tantrums, and aggression (Eyberg, 1988). PCIT is delivered in two treatment phases: (1) enhancing positive parenting skills and implementing selective attention during the Child-Directed Interaction (CDI) phase and (2) the Parent-Directed Interaction (PDI) phase emphasizing the introduction of structured discipline (e.g., removal of a privilege) within the context of continued CDI-skills. However, research has shown that the PDI phase may not be necessary for treatment success (Lieneman et al., 2017). As part of the phased intervention, parents are taught skills in each phase and then assisted in changing their parenting behavior via direct coaching strategies from a therapist to shape the child’s behavior to decreased externalizing difficulties through reinforcement. PCIT is strongly rooted in social and developmental theories employing behavior management strategies that include rewarding prosocial behavior, ignoring inappropriate behavior, and consequences for poor behavior (Eyberg, 1988). The intervention also emphasizes increasing positive interactions between a parent and child by increasing praise, special time together, and enjoyment within the interaction. In addition to behavioral principles, PCIT also has some foundations in attachment theory. For example, PCIT emphasizes the development of a strong parent–child relationship as well as contingent and sensitive responding on the part of the parent during interactions (Allen et al., 2014). PCIT has demonstrated its efficacy across several randomized controlled trials and populations (Thomas and Zimmer-Gembeck, 2007, 2011; Allen et al., 2014; Lieneman et al., 2017), demonstrating large effects sizes with regards to child and parent behaviors.

Despite supporting parents to meet some of their child’s attachment needs (e.g., contingent responding and sensitive caregiving), there are some PCIT procedures that are misaligned with attachment-based approaches which emphasize timely responsiveness to child behavioral cues as well as viewing child behaviors as reflecting an underlying need, particularly for children with a history of maltreatment. Specifically, due to the nature of miscuing, reinforcement strategies are likely less effective as the desired behavior (e.g., asking for help) may have a low base rate because the child may not view the parent as a resource. Secondly, behavioral miscues may mask underlying emotional-regulation needs, as such parental attempts to co-regulate the underlying emotion may offer an opportunity to build skills in contrast to withholding attention (Holden et al., 2022). Third, parents of maltreated children may require additional support to take charge when challenging behaviors arise, which is emphasized in COS-P. Using an intervention that provides the parent with a framework for why and how to repair the relationship with their child has the potential to enhance parent engagement and ultimately treatment outcomes of PCIT, particularly for families who have experienced maltreatment.

### 1.2. Circle of Security-Parenting

There has been significant progress over the last 20 years on the development and evaluation of attachment-based parenting programs (Gregory and Sharman, 2020). The Circle of Security-Parenting program (COS-P); (Cooper et al., 2009). COS-P is a revised 8-session program of the original 20-session Circle of Security (COS) intervention. The COS-P program was adapted to be more cost-effective, brief, and scalable for implementation (Cooper et al., 2009). The goals of the COS-P intervention are to increase a parent’s ability to recognize their child’s needs, increase sensitive parenting behavior, increase parent emotion regulation, and decrease negative attributions toward the child by the parent (Cooper et al., 2009). Using decades of attachment research, the COS-P program is facilitated by a trained provider who uses computer-delivered content, parent reflection, and discussion, to teach parents core concepts of attachment, intergenerational trauma, regulating emotions, and how to identify and more consistently meet their child’s needs to improve the parent–child relationship.

Randomized-controlled trials of the COS-P program have demonstrated modest results with regards to child behavior (Cassidy et al., 2017; Zimmer-Gembeck et al., 2022). One hypothesized reason for a lack of effect on child outcomes is that while parents understand the concepts of attachment following the COS-P intervention, they may lack sufficient opportunities to apply and practice skills in a structured way. That is, COS-P lacks the *in vivo* practice component that is a pillar of the PCIT intervention. Thus, providing parents with an initial attachment framework to understand and to increase motivation for treatment, followed by the practice component of PCIT may be a particularly fruitful way to decrease attrition and improve child behavior difficulties following maltreatment. An understanding of an attachment framework may also increase the mastery of PCIT skills among caregivers due to knowing *how* these behaviors may support their child’s needs and decrease aggressive behavior. Below, attachment theory as presented by COS-P will be discussed and used as a framework to understand the attachment-based elements of PCIT.

The COS-P program helps parents understand attachment theory with the use of a graphic that highlights important theoretical concepts and the role of the parent (Marvin et al., 2002; Maxwell et al., 2021) (The graphic is available at https://www.circleofsecurityinternational.com/circle-of-security-model/what-is-the-circle-of-security/). The graphic illustrates that the parent serves as the basis for exploration and connection processes. For this system of exploration and connection to work optimally, the child expresses their attachment needs directly, increasing the likelihood that the parent will acknowledge or respond to these needs. When a child is unable to express an attachment need directly, then the child may miscue (e.g., a child may be disruptive or aggressive rather than seeking help or expressing an emotional need). The theory posits that these miscues are shaped by the distress experienced by the parent, and then shared by the child, in response to meeting a particular attachment need. Over time, the child learns to avoid the compounded distress of expressing this need directly and adopts a miscue (i.e., aggressive behavior). Given that the child’s miscue is rooted in parental discomfort, through COS-P, the parent learns that the parent is well-positioned to be the agent of change in the relationship by inviting new patterns of interaction (i.e., direct expression of attachment needs). Further, COS-P offers parents tools to identify and understand their distress, identify their child’s attachment needs, the conditions under which their children can express their attachment needs directly and a framework for parents to understand their own emotional regulation in the service of co-regulating their children’s emotions. After the program, parents view behavior as an expression of an underlying attachment need that the parent can address if the parent acknowledges and manages their distress in the moment or by engaging in relational repair.

Further, the graphic organizes and illustrates that children may experience specific attachment needs within the relationship and that miscues may be associated with three aspects of the Circle of Security graphic. First, when a child is engaging in exploratory behavior, children need the parent to watch over them, to delight in them, to enjoy with them and to help them. Second, when a child is engaging and connecting behavior, in addition to delight, children need the parent to protect them, to comfort them, to welcome them and to assist with emotional co-regulation. Third, the child requires the parent to be present and demonstrate kindness alongside the ability to take charge when necessary. This parental balance is a necessary condition for the exploratory and connection attachment needs to be expressed directly. Importantly, the intervention describes how longstanding parental distress can result in children miscueing their attachment needs associated with exploration, connection and/or having a parent to anchor these processes (i.e., taking charge in a kind way). As such, parents have a clear explanation of why the disruptive behavior is occurring (i.e., miscues), what needs to change for the child to be able to express their attachment needs directly and that the change process will cause some distress.

### 1.3. Enhancing attachment-based aspects of PCIT

Examining PCIT from the framework of COS-P, the attachment-based elements of PCIT are readily identified in the Child Directed Interaction (CDI) phase but this examination reveals aspects of attachment theory that are not emphasized in the PCIT model (see Figure 1, Panel **B** for strategies and relational conditions present in PCIT). The CDI phase involves child-directed play which emphasize the exploratory attachment needs. Indeed, the caregiver skills acquired in CDI implicitly communicate that the **parent is actively available to the child** to meet these attachment needs (by commenting their observations and delighting through praise). In contrast, PCIT does not appear to have coaching strategies designed to address connection needs (e.g., emotional co-regulation, protection, and comfort). The need to bolster the emotion-regulation skills of the dyad has been recognized in the PCIT literature both in recent research and in descriptions of clinical practice (Campbell et al., 2023). As such, the parent may need to be coached to be explicit about the child’s internal states mattering to the parent and that emotional expression is permissible in the relationship (i.e., **parent open to emotion**). Additionally, given that the parent may not have been available historically, additional coaching strategies may be necessary for children to cue their needs directly, such as COS-P’s empathic process of “**being with**.” The child may benefit from direct statements of availability from the parent and explicit repair attempts.

Examining the Parent-Directed Interaction (PDI) phase of PCIT from an attachment perspective, parents may need additional support to lead and take charge, particularly for those who have been emotionally and/or physically unavailable due to addictions, mental health concerns and/or domestic violence. For parents who have historically struggled to take charge, it is important to develop the ability to take charge in a way that minimizes the parent’s distress. When a parent takes charge in a way that aligns with the child’s attachment need, this approach may avert additional distress related to limit setting and be experienced as more kind, allowing parents to **take charge in a kind way**. Theoretically, these efforts should occur early in treatment to bolster the parent’s active invitations for children to express their exploratory and connection attachment needs directly as the parent establishes themselves as the anchor for this process.

## 2. Attachment-based enhancements to Parent–Child Interaction Therapy

There are four main reasons for enhancing the attachment-based concepts within the PCIT intervention: (1) increasing parent motivation, engagement, and retention, (2) building parental understanding and appreciation for the relational conditions required for a child to express a miscued need directly, (3) using coaching strategies to make the implicit aspects of PCIT (such as the **parent available to the child** and the emotional regulation aspects) more explicit, and (4) highlighting the ability for the parent to take charge in a kind way from the outset of treatment by aligning with the child’s needs.

Figure 1, Panel **B**, depicts the conceptual process by which enhancing the attachment components of PCIT with COS-P can lead to a shift in child behavior. Specifically, increased awareness of attachment principles paired with relational strategies and PCIT skills, lead to establishing relational conditions that promote changes in child behavior. If a relational condition is not met, then children miscue rather than engage in an adaptive behavior. As such, direct cues around attachment needs (e.g., asking for help) may occur so infrequently that these behaviors are not amenable to reinforcement strategies, requiring the parent to actively invite these direct cues. Because of their own experiences of child maltreatment, intimate partner violence, or mental health difficulties, parents of maltreated children often struggle with the relational conditions that support children in expressing their needs in adaptive ways (Cohen et al., 2008). Thus, parents of maltreated children often require additional support to establish the relational conditions for PCIT to be successful.

In the attachment enhancement to PCIT, parents first complete the COS-P intervention to increase their understanding and their motivation to learn what is required for children to cue their needs more directly (e.g., relational conditions). Subsequently, parents apply their knowledge and identify the relational conditions (identified in bold above and presented in Figure 1, Panel **B**) that require additional support in conjunction with their therapist. Parents complete a didactic session where they learn the CDI skills from PCIT and the relevant Relational Conditions strategies (RC strategies) derived from the COS-P intervention (see Table 1). Then, RC strategies are coached alongside CDI skills to foster the necessary relational conditions. Once the relational conditions are established for the family (i.e., the caregiver is routinely using the RC strategies which are leading to increased child communication of emotions and needs), the child’s direct cues are reinforced through traditional PCIT strategies and RC strategies continue to be integrated to create a lasting shift in the child’s behavior. The child’s responses to the caregiver’s use of RC strategies are documented along with PCIT behaviors following coaching sessions. Next, we discuss in detail each component of the attachment enhancements to PCIT.

**Table 1:** Description of Relational Condition strategies incorporated into the PCIT approach.

Skills for establishing the four relational conditions	Description
***Taking charge in a kind way***	
Giving permission (relationship)	To establish that the parent can take charge, when necessary, the idea of giving permission for behaviors that align with the child’s current need is introduced. For example, if the child starts to leave the table, the parent is coached to say: “I give you permission to leave” to introduce and to reinforce the concept of parent control. Similarly, some PCIT skills can be modified to include the word “allowed” to establish parental oversight in alignment with the child’s need. Over time, this approach evolves into supporting the child’s actions of waiting for permission or asking for permission by applying the traditional PCIT skills.
Advocacy (Relationship)	When advocating, the parent is taking charge in a way that aligns with the child’s need by requesting something for the child from another adult. This strategy informs the child that a parent can recognize a need and help the child with this need so that the child views the parent as a capable resource. The most effective advocacy strategies allow the child to witness the parent advocating for the child’s need directly. Initially, this occurs in the session with the therapist, but our most successful cases have involved parents who use this approach outside the session.
***Parent available to child***	
Statement of availability (relationship)	To establish that the parent is available to the child, the parent is coached to make explicit statements about their availability (e.g., “I am here to help you”)
Ascribing good intent (relationship)	When ascribing good intent, the parent comments on a neutral behavior and uses PCIT skills to reinforce a desired behavior. For example, “Thank you for thinking about how I can help you.” This strategy has been very successful with children who tend to freeze or whose problematic behaviors are conceptualized as miscues.
Delight (relationship)	This core concept from the COS-P resonates with the PCIT skill of Enjoy. It refers to behaviors that show the child that the parent is enjoying the interaction and that the child is worthy of love, attention, and engagement. Delight behaviors may include positive affect, a warm gaze, mutual smiles, or shared laughter.
Repairing (relationship)	A parent asserts their availability when they return to a situation where they were unavailable and attempts to repair this rupture with the child. For example, “I think you were trying to tell me something important and I did not understand. I’m sorry. Together we’ll figure out what you were trying to tell me.”
***Parent open to emotion***	
Co-regulation (parent)	The parent is encouraged to regulate themselves (i.e., take deep breaths) and to provide physical and emotional comfort to their child when distressed. Through COS-P, parents have often identified the specific emotions that they avoid, and they are supported in tolerating these emotions in themselves and their children.
Permission for emotion (relationship)	By modelling their own verbal expression of low-level emotion, the parent demonstrates to the child that emotional expressions are acceptable to express in the relationship. For example, when a play session is ending, the parent is coached to say: “I am sad to be ending our special play time together.” This message introduces permission for a broader range of shared emotions, and alongside other strategies, gives the child permission to express their feelings as well. Through COS-P, parents have often identified the specific emotions that have been avoided within the historical context of the relationship and this strategy if often necessary to shift this pattern. In our experience, cases where there is an avoidance of joy require extensive use of this strategy, as this intolerance hamstrings many of the PCIT skills.
Communicating internal states matter (relationship)	The parent is coached to use PCIT skills to reinforce any instance of a child sharing an internal state (e.g., thoughts or plans). For example, “I really like when you tell me what you want to do next.” In our experience, the reinforcement of thoughts is followed by bridging statements (“It is so fun to play with you when you share your plans and your feelings”) prior to seeing any direct expression of emotion from the child.
***“Being With”***	
Tolerating parental distress and identifying miscues (parent)	Especially, when the child is starting to get angry or show signs of aggressive behavior, the parent is encouraged to notice their own distress and activation and to acknowledge the child’s behavior as a miscue while being supported to remain present in the interaction.
“Being with” (relationship)	To build the relational capacity of being with, the parent must try to “be with” their child across a range of emotions. Parents are coached to be attentive to body language and facial expressions and to make empathic statements intended to show the child that the parent can tolerate the feeling and therefore support the child with co-regulation. As part of teaching this strategy, we warn parents that the child may respond with “freezing.” The use of emotional labels is usually coached later in the process with initial coaching focused around supporting children in “showing” their feelings and providing explanations to organize their feelings (e.g., “It is so hard when you have a plan, but it does not seem to be working the way you want”).

### 2.1. Parent awareness and motivation to change

Parents of children who have been maltreated can benefit from COS-P psychoeducation to understand their child’s disruptive behavior as miscues, their responses to the behavior, and the relational patterns that occur. This understanding provides the underlying rational for coaching the RC strategies. During COS-P, the parent also becomes aware of their own distress related to meeting their child’s attachment need that may limit their ability to take charge or respond with empathy (i.e., “being with”). By understanding *why* the child is miscueing with aggressive behavior and the role of relational patterns, parents become engaged and motivated in the treatment (See Figure 1, Panel **B**). For example, rather than responding to aggressive behavior with a punishment or privilege removal, the parent can identify the behavior as a need and take steps toward meeting the need. Lastly, through the COS-P program, the parent learns that there is often discomfort in shifting longstanding relationship patterns and that the ability to acknowledge this distress is often necessary for treatment progress.

Through COS-P (typically delivered in an 8-week group setting, offered virtually), parents develop an attachment-informed understanding of their child’s concerning behaviors, learn to reflect on their own responses to their child’s behavior, and appreciate how their previous interactional patterns were maintaining the child’s behavior difficulties. Using the COS-P framework, parents realize that they may be limited in meeting their child’s emotional needs in the past due to preoccupation with family violence or other adverse events. In addition to limited availability, many parents also realize their struggle to **take charge in a kind way** or to respond with empathy (“being with”) and how these fundamental limitations undermine their relationship with their children. Through this process, parents learn that their child does not perceive them as an available resource to support their emotional needs. In our experience, parents leave COS-P with a hopeful, non-defensive and accurate description of *why* changes are necessary and *what* needs to change in their relationship with their child, embracing the idea that they are the agent of change in this process.

### 2.2. Coaching of COS-P and PCIT skills

Prior to the coaching sessions, the parent and the therapist set goals related to the behavioral shift the parent would like to see in the child (e.g., using their words to express their needs directly rather than be aggressive). In PCIT, this collaboration is followed by teaching the parent how to use CDI strategies that will increase the child’s desirable behavior (e.g., verbalizing anger as opposed to acting aggressively). We follow this process and, with a shared knowledge of COS-P, the therapist and the parent efficiently discuss the relational conditions that may be interfering with the child’s ability to express their emotions directly to the parent (i.e., parent open to emotion, being with, parent available to child, and taking charge in a kind way) (See Panel **B** of Figure 1). Based on the outcome of this discussion, the applicable Relational Conditions strategies (RC strategies) are taught (see Table 1). These RC strategies include concepts from the COS-P program (e.g., “being with”) and new skills we created to translate COS-P attachment theory into coachable concepts (e.g., permission for emotion, communicating internal states matter). That is, the treatment goals include fulfilling these relational conditions so the child may learn to express emotions directly rather than miscuing with aggression.

Through coaching, parents learn to apply the CDI skills and RC strategies simultaneously. Parent–child interactions are coached individually during a play session, and the coaching can be done in person or virtually. If done virtually, the parent sets up a camera that captures the parent–child interaction using a secure web-based platform. In either format, the parent wears a headset so that they can receive instructions and support from the therapist. Parents can receive between 5 and 10 coaching sessions for the treatment goal of eliminating physical and verbal aggression toward a parent.

Throughout the coaching sessions, there are three main components: traditional PCIT skills, new relationship-focused RC strategies, and new parent-focused RC strategies. The relationship-focused component shapes new interactions between the parent and the child based on an understanding of how the relational conditions may be limited while the parent-focused component provides direct coaching and support related to the parent’s self-regulation and emotional needs. The RC strategies associated with each relational condition are summarized below and described in Table 1 with an indication of whether the strategy is parent or relationship focused.

The RC strategies assist parents in establishing the four relational conditions (bolded below). As collaboratively established in goal setting, a parent **takes charge in a kind way** by using strategies that align with the child’s need, such as giving permission and advocacy, within the context of child-led play. Given the theoretical significance, this relational condition is always prioritized. The other required RC strategies are implemented in treatment. For the **parent to be available to the child**, the parent is coached to overtly communicate their availability, ascribe good intent to the child’s behaviors and make active repair attempts when they have not been available. In fulfilling **parent open to emotion**, parents first establish that internal experiences (e.g., thoughts, memories, and plans) can be shared with the parent and, then that emotions are also permissible by making direct statements or modelling low intensity expression of emotion. For example, to give permission for emotion, a parent is coached to state that the parent is sad or angry that the play session is over, providing a brief rationale to self-validate this feeling. Once more comfortable with these interactions, bridging statements that invite direct emotional expression from the child are coached to expand the range of permissible emotions displayed by the dyad. Given that parents are often able to **be with** some emotions, they are encouraged to **be with** feelings within their comfort zone and then are coached to expand their emotional repertoire, providing the safety of emotional co-regulation to their child.

Although the setting of limits, ignoring of inappropriate behavior, and consequences are usually implemented in PCIT, the RC strategies early in the intervention often make this aspect of treatment unnecessary. The treatment appears streamlined as the frequency and intensity of undesirable behaviors appear to be lessened. After completion of CDI, an assessment informs whether the family requires additional support with PDI. This additional phase is provided to most maltreating caregivers and most caregivers with a history of domestic violence. This PDI phase tends to be brief and uneventful. This approach is consistent with the findings that PCIT can be effective in realizing desired behavioral outcomes without the implementation of PDI (Thomas and Zimmer-Gembeck, 2007) and a clinical practice described by Campbell et al. (2023).

### 2.3. Relational conditions

By supplementing the PCIT skills with RC strategies, the relational conditions that were challenged in parent–child dyads exposed to maltreatment can be bolstered. This step in the process is based on the accumulation of experiences that create a change in the relationship: over time, the relational condition is met by using the RC strategies.

### 2.4. Child behavior shift

Once the parent establishes the necessary relational conditions, the child is more likely to directly cue their attachment need and PCIT and RC strategies can be effectively used to support the shift in the child’s behavior. Gradually, the child learns that their parent is available to them and is an effective resource. Then the child can risk cueing their needs directly aligned with treatment goals. The goal is for these new relationship patterns to gain momentum and be sustained after treatment.

## 3. Recommendations for implementation

In our experience using the attachment enhancements to PCIT with a range of caregivers (e.g., biological parents, kinship parents, and grandparents) presenting with primary maltreatment exposures (e.g., sexual abuse, physical abuse, or both), families have been successful in completing PCIT treatment in approximately 4 to 16 sessions. This range of sessions exclude the COS-P group sessions and only include the PDI phase of PCIT when indicated. Since the implementation of this approach, no families have prematurely dropped out from treatment and all families have reached their identified goal of reduced child disruptive behavior and aggression. The enhanced attachment-based approach can be delivered in-person and virtually by clinicians trained in both PCIT and COS-P. Training in both modalities is necessary to understand key concepts and mechanisms of change for both approaches.

## 4. Limitations

The current attachment-based enhancement to PCIT includes some limitations. First, to provide the adaptation, the therapist must be trained and well-versed in both PCIT and COS-P. This training presents challenges as obtaining training for both modalities can be costly and is intensive for community-based practitioners. A second limitation is that although we have documented the treatment completion of those who have received the adapted intervention, we have yet to conduct a formal evaluation of the outcomes and as such our results should be considered very preliminary. Thus, to what extent outcomes differ between families who receive PCIT alone and those that receive the attachment-based enhancement to PCIT remains to be investigated.

## 5. Conclusion and future directions

Enhancing a well-established parenting program, Parent–Child Interaction Therapy (PCIT), with the delivery of an attachment-focused intervention (Circle of Security Parenting) and additional coaching strategies has the potential to increase parent engagement and decrease attrition for parent–child dyads exposed to maltreatment. A formal evaluation of quantitative changes in child behavior difficulties following the adapted PCIT intervention are needed to demonstrate the effectiveness of the intervention. A randomized controlled trial comparing engagement, retention, and both child and parent treatment outcomes for individuals who receive standard PCIT and those who receive the attachment-based adaptation is also needed. Future research should also investigate the long-term follow-up of children who receive the adapted intervention to see if treatment effects are maintained over time.

## Data availability statement

The original contributions presented in the study are included in the article/supplementary material, further inquiries can be directed to the corresponding author.

## Author contributions

KB, HG, NO, and NR contributed to the intellectual content of the manuscript, drafted the manuscript, edited, and revised all the work. All authors contributed to the article and approved the submitted version.
